# A novel long noncoding RNA AC092718.4 as a prognostic biomarker and promotes lung adenocarcinoma progression

**DOI:** 10.18632/aging.204426

**Published:** 2022-12-07

**Authors:** Siqin Chen, Yang Yu, Yixiao Yuan, Xi Chen, Fan Zhou, Yongwu Li, Ping Wang, Xiulin Jiang, Sha Tian, Wenjun Ren

**Affiliations:** 1 Department of Oncology, Affiliated Hospital of Hunan Academy of Traditional Chinese Medicine, Changsha 410006, Changsha, China; 2 Department of Thoracic Surgery, The First People’s Hospital Yunnan Province/The Affiliated Hospital of Kunming University of Science and Technology, Kunming 650034, Yunnan, China; 3 Key Laboratory of Molecular Oncology and Epigenetics, The First Affiliated Hospital of Chongqing Medical University, Chongqing 400016, Chongqing, China; 4 Department of Neurosurgery, The Second Affiliated Hospital of Kunming Medical University, Kunming 650223, Yunnan, China; 5 Department of Cardiothoracic Surgery, The First People’s Hospital of Yunnan Province/The Affiliated Hospital of Kunming University of Science and Technology, Kunming 650034, Yunnan, China; 6 Department of Thoracic Surgery, The Second Affiliated Hospital of Kunming Medical University, Kunming, China; 7 College of Life Science, University of Chinese Academy of Sciences, Beijing 100049, China; 8 Department of Internal Medicine, College of Integrated Chinese and Western Medicine, Hunan University of Chinese Medicine, Dr. Neher’s Biophysics Laboratory for Innovative Drug Discovery, State Key Laboratory of Quality Research in Chinese Medicine, Macau University of Science and Technology, Taipa 410006, Macau, China

**Keywords:** lncRNA, lung adenocarcinoma, immune cell infiltration, prognosis biomarker, cell growth

## Abstract

Long noncoding RNAs (lncRNAs) reportedly play critical roles in the pathogenesis of various cancers, including lung adenocarcinoma (LUAD). However, the expression level, clinical significance, and potential function of lncRNA-AC092718.4 in LUAD remain unclear. In this study, we found that AC092718.4 was highly expressed in LUAD and high expression of AC092718.4 was correlated with poor overall survival (OS) and disease-specific survival (DSS) in LUAD. Cox regression analysis confirmed that AC092718.4 was an independent factor for LUAD prognosis. Kyoto Encyclopedia of Genes and Genomes (KEGG) results showed that AC092718.4 was involved in the PI3K-Akt signaling pathway, Th17 cell differentiation, and cell apoptosis. AC092718.4 expression was correlated with immune cell infiltration. Finally, we found that the knockdown of AC092718.4 inhibited lung adenocarcinoma (LUAD) cell growth and promote cell apoptosis. Our findings confirmed that AC092718.4 may serve as a potential prognostic biomarker in LUAD.

## INTRODUCTION

Lung cancer poses a great threat to people’s health and creates a great economic burden on society. At present, there are no absolute cures for cancer. The majority of patients eventually die of cancer metastasis, recurrence, or complications. With the development of diverse methods, searching for potential biomarker cancer diagnosis and treatment has become more accessible. Lung adenocarcinoma is a type of NSCLC, and most affected patients are in an advanced, inoperable stage [1]. Therefore, determining the effective biomarker is crucial for the treatment of LUAD patients.

Emerging studies indicated that lncRNAs were crucial for human system development and cancer progression [2]. Increasing evidence has illustrated that lncRNA abnormal expression is involved in cancer progression. For instance, it has been confirmed that lncRNA-FEZF1-AS1 is elevated in lung cancer and promotes lung cancer development via activating the WNT pathway [3]. However, information available on levels, regulations, and significances of AC092718.4 in LUAD have remained lacking.

Our study is for the first time to analyze the role of AC092718.4 across diverse cancer types. The expression levels of AC092718.4, its correlations with diverse clinical features, prognosis, immune system, and its potential molecular functions and mechanisms in pan-cancer were systematically accessed through public databases. We further confirmed the up-regulation of AC092718.4 in LUAD and its promotion effect on cell proliferation in LUAD. In summary, AC092718.4 has the potential value as a biomarker for determining prognosis in a variety of cancers.

## MATERIALS AND METHODS

### Down lung cancer data

We obtained the expression data and clinical information on lung cancer from the TCGA database (https://portal.gdc.cancer.gov/). In this finding, TCGA datasets utilized in the analysis of the prognosis and diagnosis events of AC092718.4 in lung cancer. ROC curve analyzed by R code.

### Immune cell infiltration analysis

The proportions of 22 immune cells infiltrating were then inferred using the CIBERSORT algorithm, and the evaluation procedure was performed using the “ggplot2” and “heat map” R packages [4, 5].

### Cell culture

Human LUAD cell lines (H1650, A549, H1975, and H1299) lines were purchased from the Cell Resource Center Affiliated with the Chinese Academy of Sciences, and cultured in RPMI-1640 medium supplemented with 10% fetal bovine serum (100 units/ml, Solarbio, Beijing, China).

### Quantitative real-time RT-PCR and siRNA molecular transfection

The primers were designed as follows: AC092718.4-F: TGTGTGCACCTGTAATCCCA, AC092718.4-R: GGATGCAGTGGTCATCGCA. AC092718.4 was silenced by siRNA oligonucleotides (GenePharma, Suzhou, China), and the sequences were as follows, siRNA: GAGCUCUAAAAUGGAGGGA. 18sRNA is used as the internal reference gene.

### BrdU assay

This assay was utilized to validate the growth ability of the cells. The transfected cells were incubated with BrdU, cell nuclei were stained with DAPI, and observed by fluorescence microscope.

### Statistical analysis

R software 4.0.3 was used in this research. P < 0.05 were considered statistically significant.

### Data availability statement

The datasets presented in this study can be found here: the TCGA database and the Genotype-Tissue Expression (GTEx).

## RESULTS

### AC092718.4 was up-regulated in human cancers

**Figure 1:**
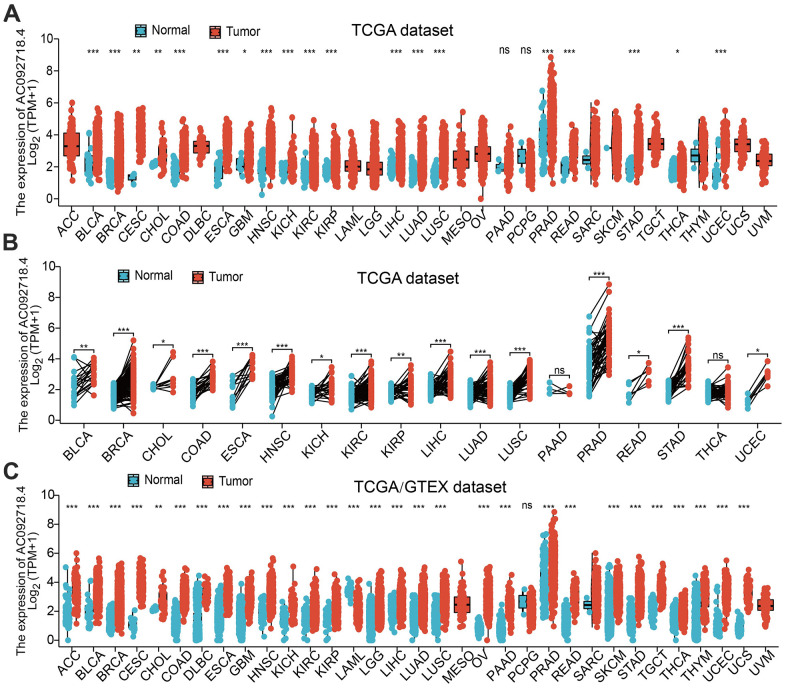
**AC092718.4 expressed differentially between tumor and normal tissues.** (**A**) The expression of AC092718.4 in pan-cancer analysis by the TCGA database (**B**) The expression of AC092718.4 in paired cancer tissues and adjacent normal tissues from TCGA datasets (**C**) AC092718.4 differential expression across different cancer types in the TCGA and GTEx databases. *P < 0.05; **P < 0.01; ***P < 0.001.

**Figure 2:**
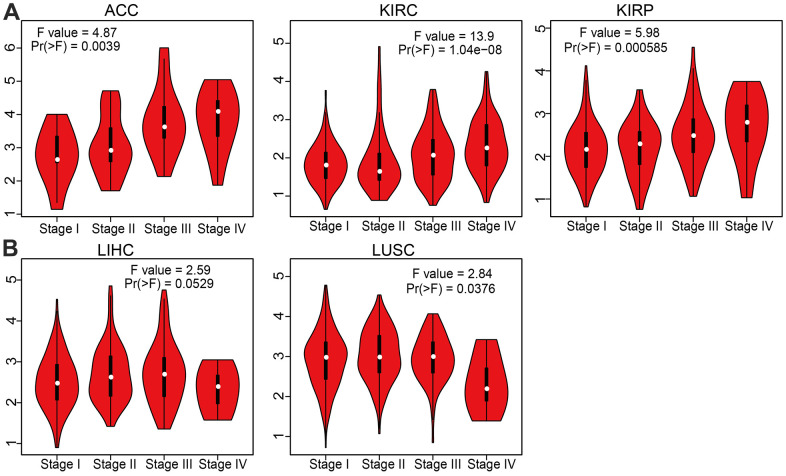
**Correlation between AC092718.4 expression and cancer stage across different cancer types.** (**A**, **B**) The correlations between AC092718.4 expression levels and tumor stage in different cancer types were examined using the GEPIA database.

In the TCGA database, AC092718.4 RNA was significantly upregulated in 19 cancers (Figure 1A). We also demonstrated that AC092718.4 was increased in 16 types of cancer than in paired adjacent normal tissues (Figure 1B). In the TCGA/GTEx database, results demonstrated that AC092718.4 expression was significantly higher in 18 types of cancer of the TCGA dataset (Figure 1C). High levels of AC092718.4 in ACC, KIRC, KIRP, and LIHC were associated with relatively poorer tumor stages (Figure 2A, 2B).

### AC092718.4 expression and patient’s prognosis

**Figure 3:**
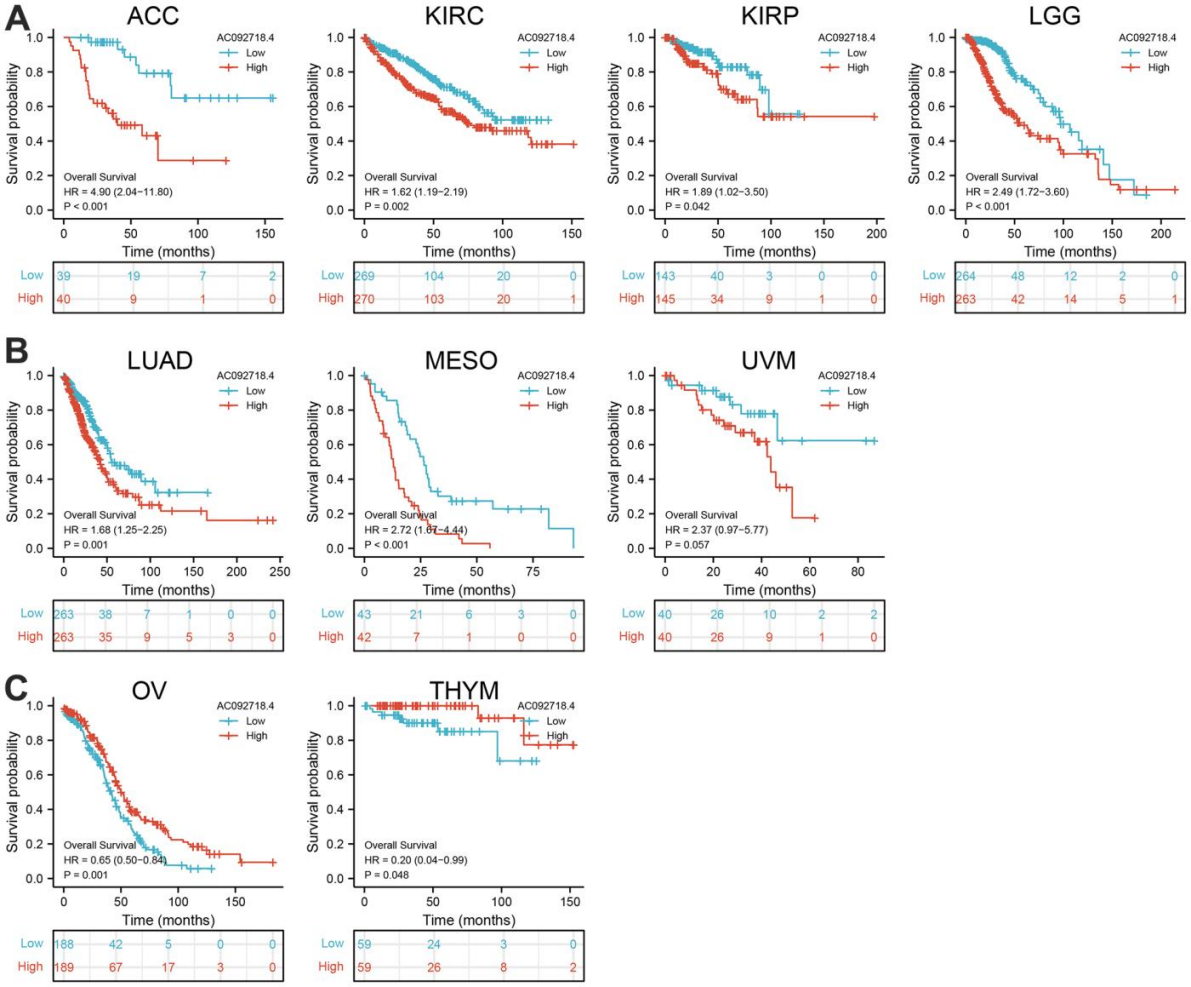
**AC092718.4 expression correlated with the overall survival of pan-cancer.** (**A**) The overall survival for AC092718.4 in ACC, KIRC, KIRP, and LGG. (**B**) The overall survival for AC092718.4 in LUAD, MESO, and UVM. (**C**) The overall survival for AC092718.4 in OV, and THYM.

**Figure 4:**
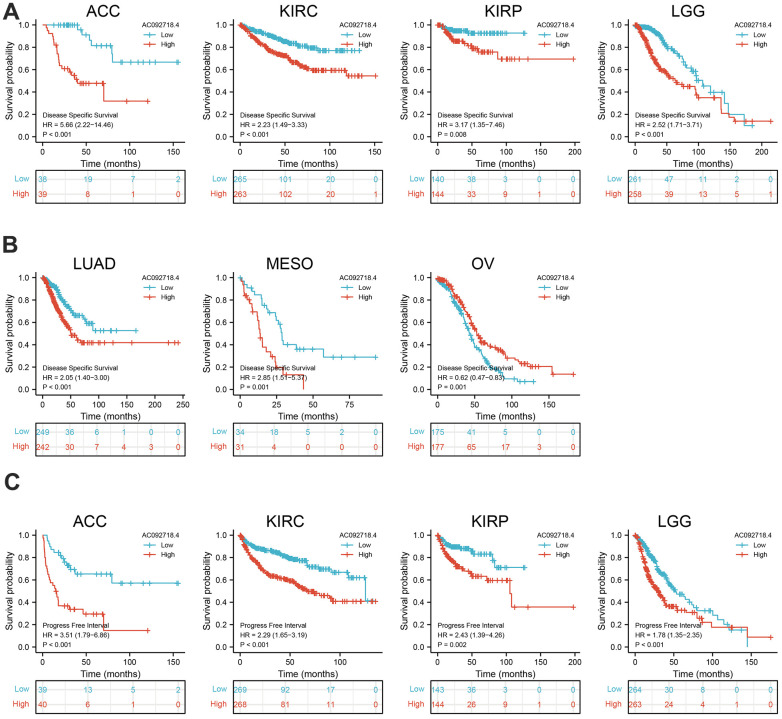
**AC092718.4 expression correlated with the disease-specific survival, and progression-free survival of pan-cancer.** (**A**, **B**) The disease-specific survival of AC092718.4 in pan-cancer. (**C**) The progression-free survival for AC092718.4 in pan-cancer.

**Figure 5:**
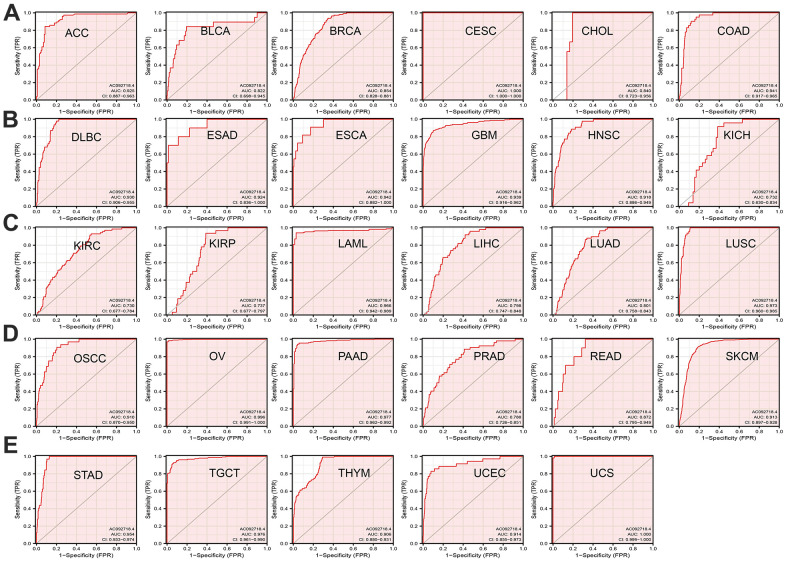
**AC092718.4 may act as a potential biomarker in human cancer.** (**A**–**E**) Predictive power for prognosis with AC092718.4 expression by ROC curve analysis in pan-cancer.

The prognostic significance of AC092718.4 in human cancer was assessed by analyzing transcriptomic data and clinical data from 33 cancers acquired by UCSC XENA. Clinical outcomes analysis demonstrated that higher AC092718.4 expression was not only correlated with adverse OS in diverse human cancers (Figures 3, 4). We also confirmed that AC092718.4 has potential diagnostic significance in human cancers (Figure 5A–5E). Collectively, these results indicated that AC092718.4 may act as a potential biomarker in human cancer.

### Molecular characteristics analysis

**Figure 6:**
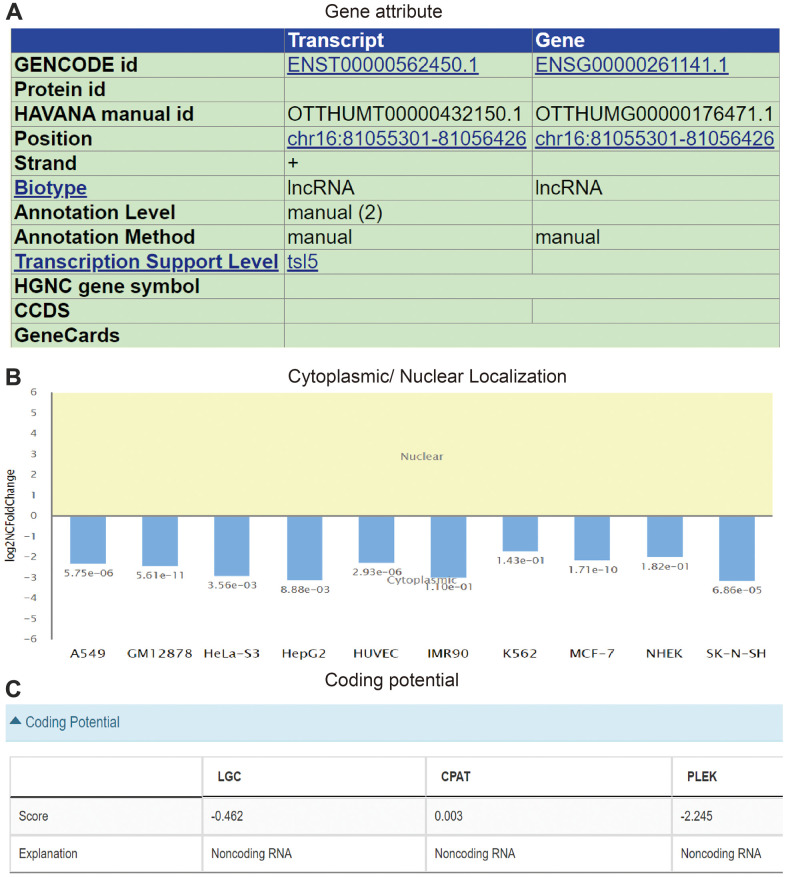
**Molecular characteristics analysis of AC092718.4.** (**A**) The genomic attributes of AC092718.4 analysis by UCSC database. (**B**) The subcellular localization of AC092718.4 in diverse cancer cells. (**C**) The coding potential of AC092718.4 analysis by LGC, CPAT, and PLEK databases.

AC092718.4 was found to be a 715 nucleotide (nt) intronless transcript that is identical to in the UCSC database, the transcript sequence shown in (Supplementary Table 1). The genomic information of AC092718.4 is displayed in Figure 6A. AC092718.4 is mainly located in chr16:81,055,301-81,056,426. Moreover, we showed that AC092718.4 is located primarily in the cytoplasm of lung adenocarcinoma cells (Figure 6B). AC092718.4 doesn’t exhibit coding potential analysis by the online database (Figure 6C).

### AC092718.4 was up-regulated in LUAD

**Figure 7:**
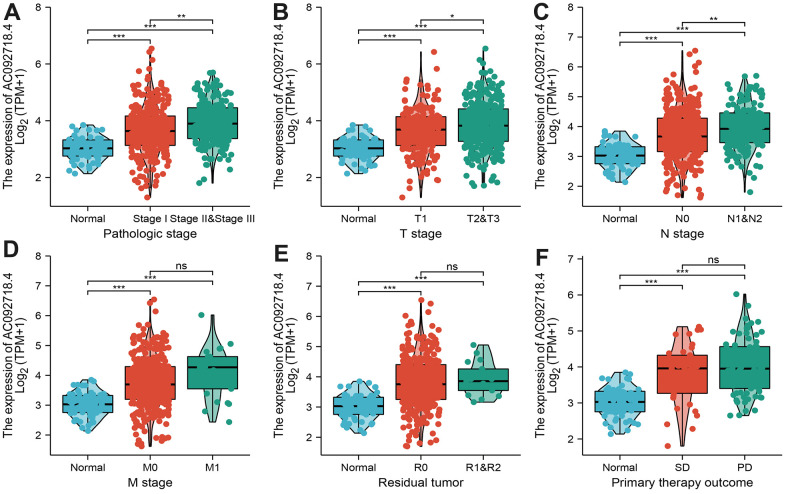
**Correlation between AC092718.4 expression and various clinical features in LUAD.** (**A**–**F**) Correlation between AC092718.4 expression and various clinical features, including pathological stage, TNM stage, residual tumor, and primary therapy outcomes.

**Figure 8:**
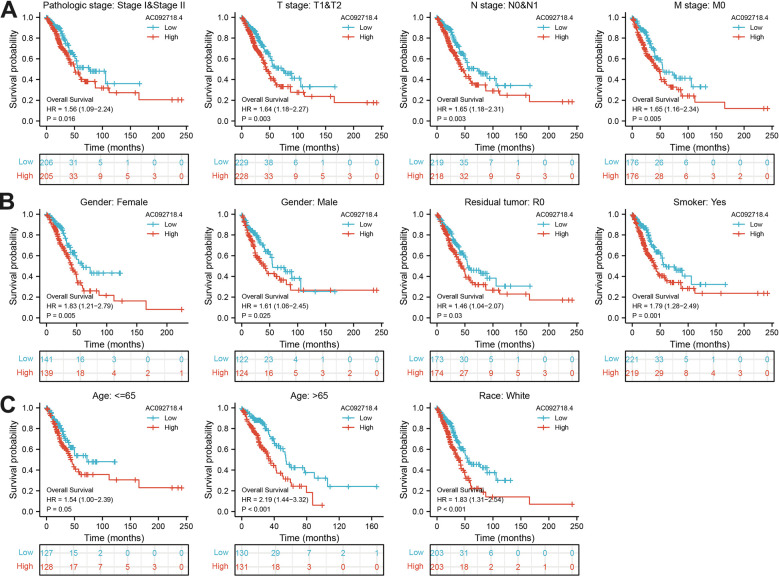
**Correlation between various clinical features and overall survival in LUAD.** (**A**–**C**) The prognosis of AC092718.4 is based on different subgroups, including stage, TNM stage, gender, residual tumor, smoker, age, and race.

**Figure 9:**
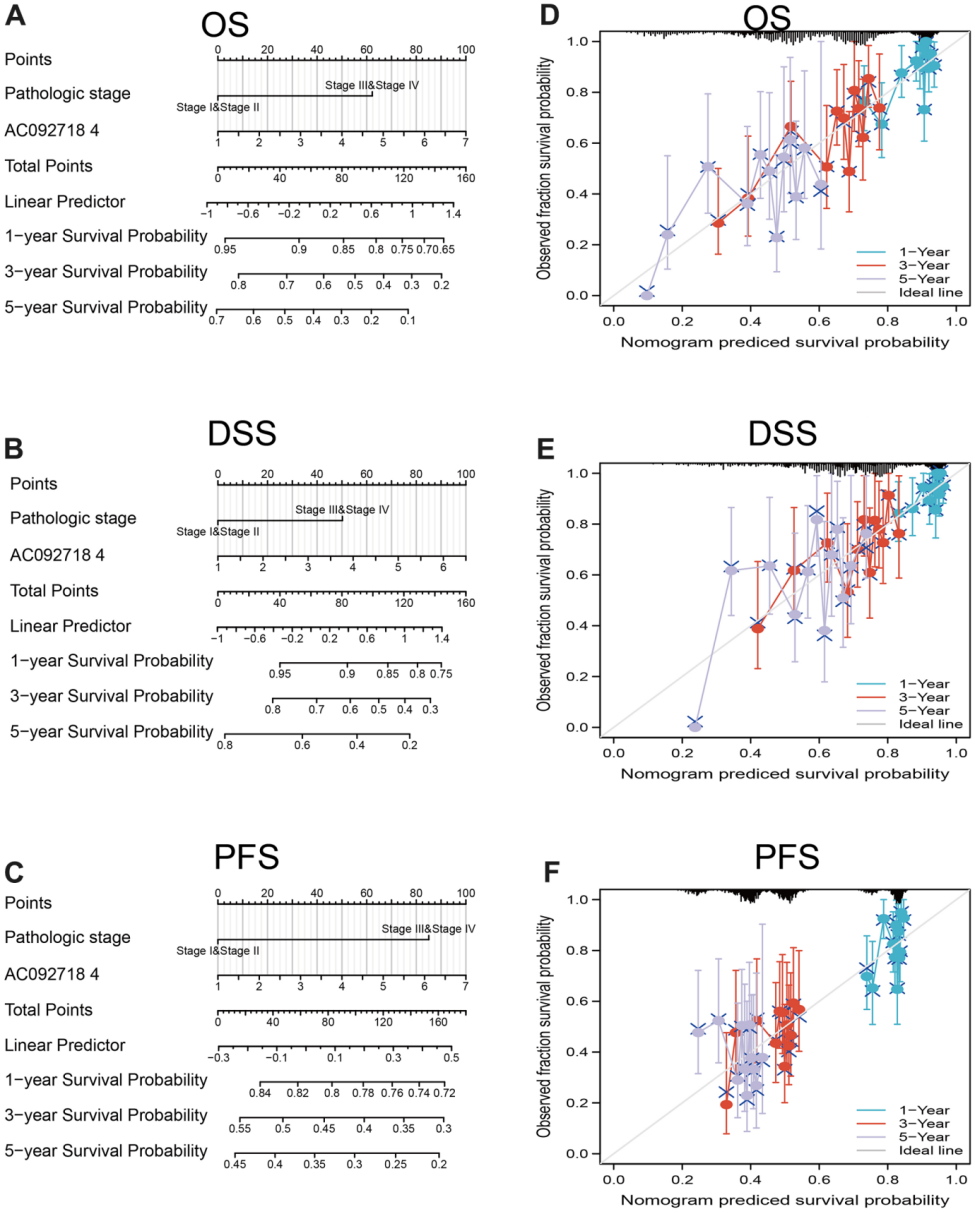
**Construction and evaluation of nomogram.** Nomogram to predict (**A**) overall survival, (**B**) disease-specific survival, and (**C**) progression-free survival of lung cancer patients The calibration curve and Hosmer–Lemeshow test of nomograms in the TCGA- lung adenocarcinoma cohort for (**D**) overall survival, (**E**) disease-specific survival and (**F**) progression-free survival.

We found that AC092718.4 expression was significantly related to the diverse clinical features in LUAD (Figure 7A–7F). Interestingly, the high expression of AC092718.4 had a poor survival time (Figure 8A–8C). The independent prognostic value of AC092718.4 in patients with LUAD was further assessed by conducting univariable and multivariable Cox regression analyses (Figure 2C). The univariable analysis showed that AC092718.4 is an independent prognostic factor for LUAD (Tables 1, 2). Moreover, we constructed a better nomogram to predict 1, 3, and 5-year survival rates of LUAD patients according to AC092718.4 expression and pathologic stage (Figure 9A–9F).

**Table 1:** Univariate and multivariate Cox regression analyses of different parameters on overall survival in LUAD.

**Characteristics**	**Total(N)**	**Univariate analysis**			**Multivariate analysis**	
		**Hazard ratio (95% CI)**	**P value**		**Hazard ratio (95% CI)**	**P value**
T stage	523					
T1&T2	457					
T3&T4	66	2.317 (1.591-3.375)	<0.001		1.855 (1.153-2.983)	0.011
N stage	510					
N0&N1	437					
N3&N2	73	2.321 (1.631-3.303)	<0.001		1.406 (0.676-2.922)	0.362
Pathologic stage	518					
Stage II&Stage I	411					
Stage IV&Stage III	107	2.664 (1.960-3.621)	<0.001		1.673 (0.771-3.628)	0.193
M stage	377					
M0	352					
M1	25	2.136 (1.248-3.653)	0.006		1.147 (0.513-2.563)	0.739
AC092718 4	526	1.317 (1.111-1.561)	0.002		1.348 (1.102-1.648)	0.004

**Table 2:** Univariate and multivariate Cox regression analyses of different parameters on disease specific survival in LUAD.

**Characteristics**	**Total(N)**	**Univariate analysis**			**Multivariate analysis**	
		**Hazard ratio (95% CI)**	**P value**		**Hazard ratio (95% CI)**	**P value**
T stage	488					
T1&T2	430					
T3&T4	58	1.974 (1.190-3.275)	0.008		1.662 (0.847-3.259)	0.140
N stage	475					
N0&N1	410					
N3&N2	65	1.971 (1.247-3.115)	0.004		1.486 (0.504-4.379)	0.473
Pathologic stage	483					
Stage II&Stage I	389					
Stage IV&Stage III	94	2.436 (1.645-3.605)	<0.001		1.348 (0.428-4.243)	0.609
M stage	344					
M0	323					
M1	21	2.455 (1.269-4.749)	0.008		1.781 (0.553-5.736)	0.333
AC092718 4	491	1.370 (1.108-1.694)	0.004		1.282 (0.993-1.655)	0.047

### GO and KEGG analyses of AC092718.4 in LUAD

**Figure 10:**
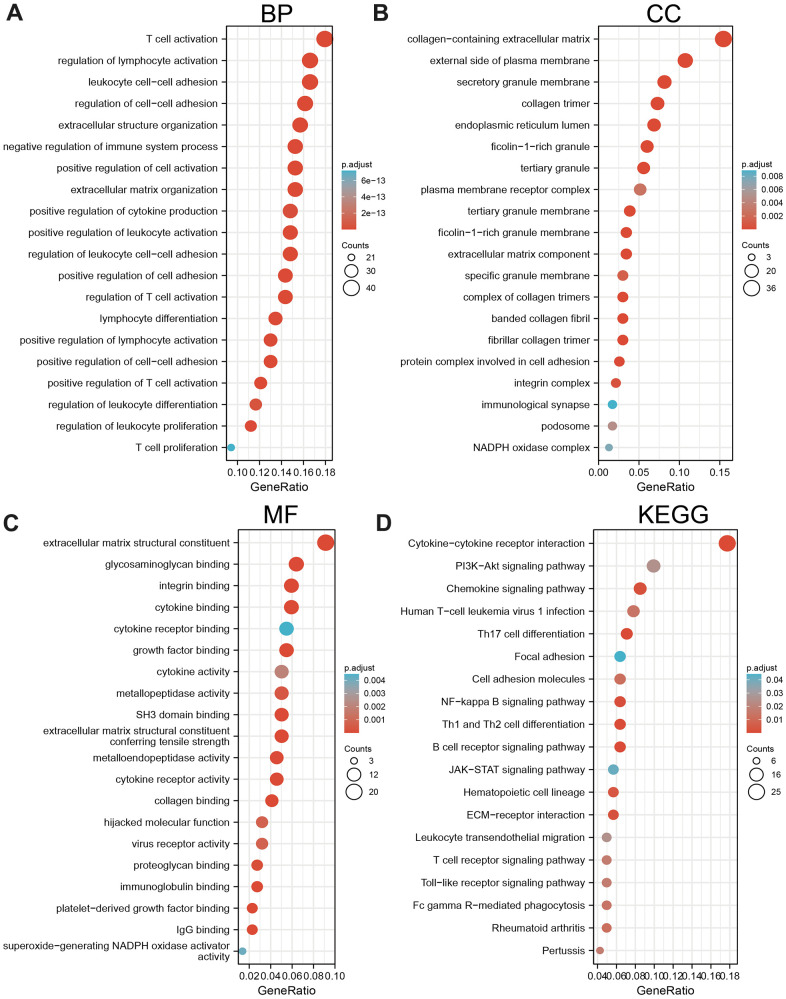
**KEGG enrichment analysis.** (**A**–**C**) GO analysis of the biological process of AC092718.4 in LUAD. (**D**) KEGG pathway study revealed that AC092718.4 was involved in the different signaling pathways.

We show the top 300 genes that are positively correlated with AC092718.4 in pan-cancer (Supplementary Table 1). The Gene Ontology (GO) enrichment analysis found that AC092718.4 was positively correlated with the immune process or immune-related pathways, including T cell activation (Figure 10A–10C). Moreover, KEGG enrichment indicated that AC092718.4 is involved in the PI3K-Akt signaling pathway, Focal adhesion, Cell adhesion, and Th1 and Th2 cell differentiation (Figure 10D).

### GSEA enrichment analysis

**Figure 11:**
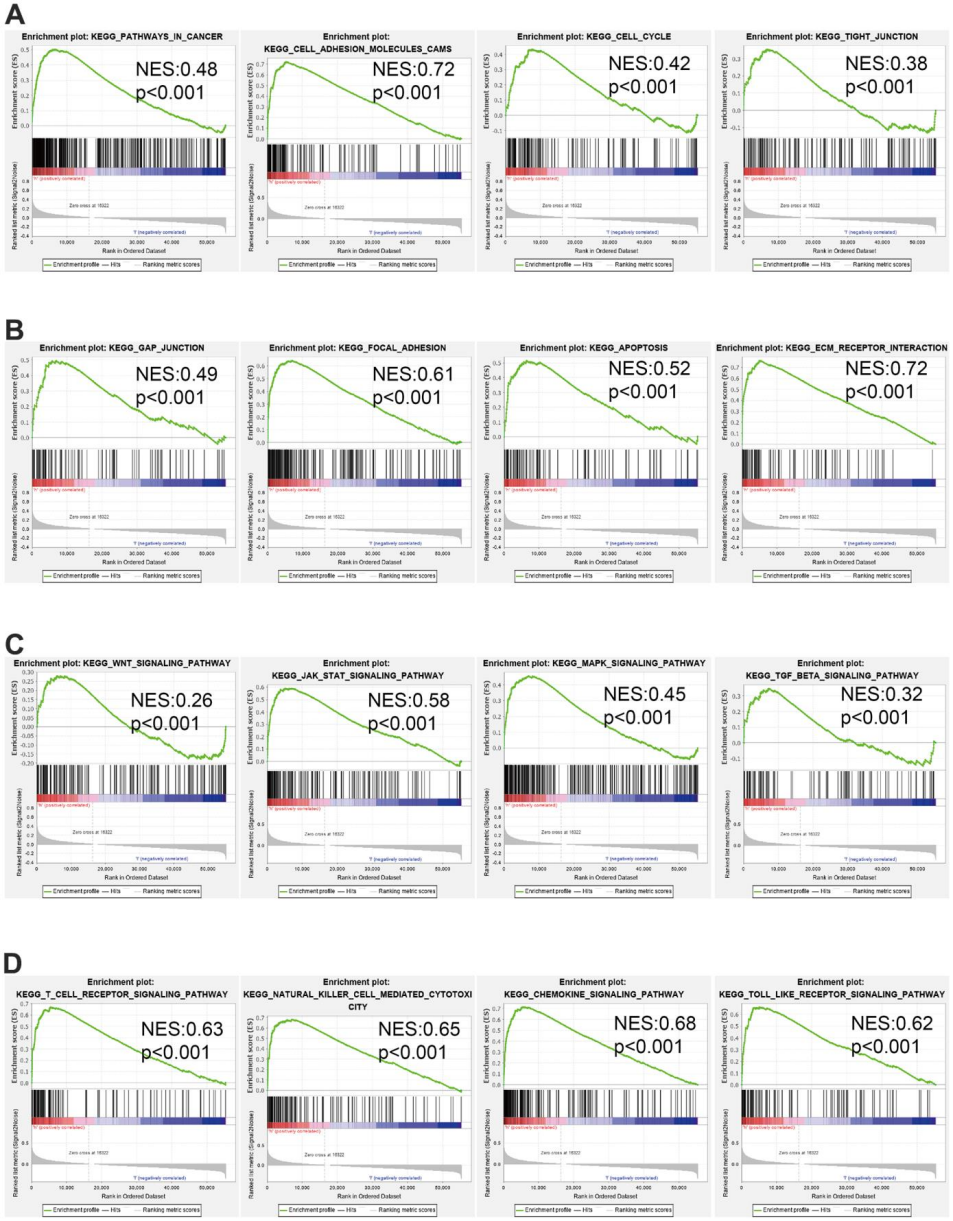
GSEA of AC092718.4 (**A**–**D**) The top GSEA results in pan-cancer. Normalized Enrichment Score (NES).

We display the positive gene with AC092718.4 in LUAD and using the above genes conducted GSEA analysis (Supplementary Table 2). GSEA analysis demonstrated that AC092718.4 mainly participated in the pathway in cancer, cell adhesion, cell cycle, tight junction, gap junction, focal adhesion, apoptosis, ECM-receptor interaction, Wnt signaling pathway, MAPK, TGF-β, Natural killer cell mediated cytotoxicity, chemokine, and Toll-like receptor (Figure 11A–11D).

### Immune cell infiltration analysis

**Figure 12:**
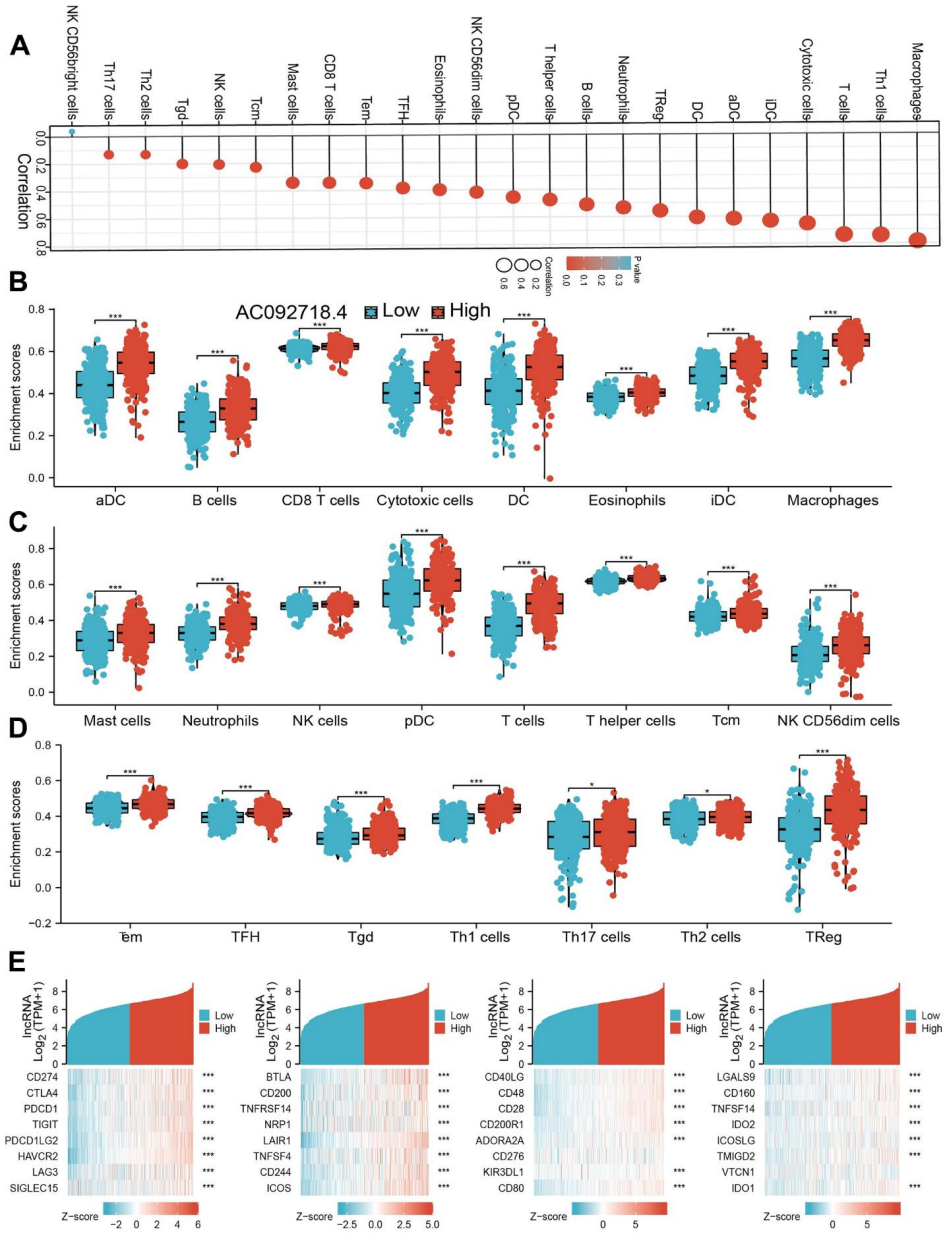
**Correlation between AC092718.4 expression and immune infiltrates.** (**A**) Correlations between AC092718.4 expression and the level of immune infiltration in LUAD using the ssGSEA method. (**B**–**D**) Correlation analysis of AC092718.4 expression and infiltration levels of immune cells in LUAD tissues. (**E**) Correlations between AC092718.4 expression and various immune checkpoint genes. *p < 0.05; **p < 0.01; ***p < 0.001.

We found that AC092718.4 high-group had a higher proportion of 22 tumor-immune cell types are shown in (Figure 12A–12D). Patients with LUAD with high AC092718.4 expression had significantly higher proportions of Macrophage, Cytotoxic cells, IDC, aDC, DC, TReg, Neutrophils, B cells, T helper cells, pDC, TFH, Tem, CD8 T cells, Mast cells, Tcm, NK cells, Tgd, Th2 cells, and Th17 cells (P < 0.05). We determine that there is a positive correlation between AC092718.4 expression and immune modulator in LUAD (Figure 12E).

### AC092718.4 knockdown inhibited LUAD progression

**Figure 13:**
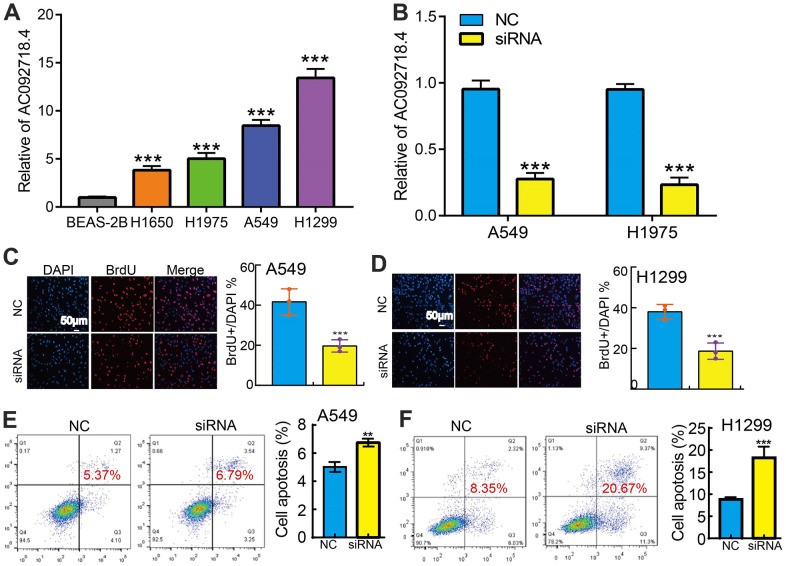
**Knockdown of AC092718.4 inhibits cancer cell growth.** (**A**) The relative expression of AC092718.4 in LUAD cell lines including H1299, H1650, A549, and H1975 examined by Real-time RT-PCR, human bronchial epithelial cells (BEAS2B) cell line was used as control. (**B**) Establishment of AC092718.4 knockdown in A549 and H1299 cells, verified by Real-time RT-PCR. (**C**–**F**) Down-regulation of AC092718.4 inhibited cell growth and promote cell apoptosis in A549 and H1299 cells. **P < 0.01; ***P < 0.001.

Our qRT-PCR results showed that AC092718.4 RNA level was significantly higher in LUAD cell lines than in normal lung epithelial (Figure 13A). AC092718.4 knockdown by siRNA and display a better knockdown efficiency (Figure 13B). We showed that the knockdown of AC092718.4 significantly decreased the growth capabilities of LUAD cells and promote cell apoptosis (Figure 13C–13F).

## DISCUSSION

It has been confirmed that lncRNAs display an important role in gene expression and various disease progression [6]. LncRNA-AC092718.4 is an identified lncRNA that is overexpressed in many cancers. However, there are no studies that confirmed whether AC092718.4 is related to LUAD progression or can be a prognostic and diagnostic biomarker for LUAD.

In this finding, we showed that AC092718.4 expression was the highest in AC092718.4 expression was significant higher in human cancers. These results confirmed that AC092718.4 may function as an oncogenic gene in human cancer. We found that higher AC092718.4 correlated with the survival times of lung cancer patients in TCGA. Higher expression of AC092718.4 related to worse clinical features and poor prognosis.

Our KEGG enrichment analysis indicated that AC092718.4 was involved in the PI3K-Akt signaling pathway, Chemokine signaling pathway, and JAK-STAT signaling pathway. For instance, LncRNA AK023391 facilitates tumorigenesis and invasion of gastric cancer by activation PI3K/Akt pathway [7]. LncRNA RP11-468E2.5 promotes colorectal cancer progression by regulating the JAK/STAT pathway [8]. The above-mentioned evidence explains, at least in part, AC092718.4 by regulating the oncogenic signaling and is involved in lung cancer progression.

The tumor microenvironment (TME) was found to be related to cancer progression [9]. In our study, we showed that AC092718.4 positively regulated the infiltration levels of macrophages, Th1 cells, T cells, Cytotoxic cells, IDC, aDC, DC, TReg, Neutrophils, in LUAD. AC092718.4 also had a positive relationship with the expression immune modulator in LUAD. Cancer cell growth and invasion usually lead to cancer progression. In our finding, we demonstrated that the knockdown of AC092718 by siRNA significantly inhibited tumor cell proliferation and promote cell apoptosis.

## CONCLUSIONS

Our study systematically analyzed the prognosis correlation between AC092718.4 and pan-cancer. The function of AC092718.4 in LUAD cancer cell lines was briefly verified by cellular experiments for the first time. However, the mechanism could not be studied and validated more deeply. The number of clinical samples was too small to perform clinical information analysis. In conclusion, AC092718.4 showed its potential as a diagnostic and prognostic marker and deserves further investigation.

## Supplementary Material

Supplementary Tables
