# Supplementary material for: A novel long noncoding RNA AC092718.4 as a prognostic biomarker and promotes lung adenocarcinoma progression
Source: Aging (Albany NY). 2022 Dec 7;14(24):9924–41. doi: 10.18632/aging.204426 (PMC9831730; doi:10.18632/aging.204426)
Supplement: Supplementary Tables [file aging-14-204426-s001.pdf]

SUPPLEMENTARY TABLES

Supplementary Table 1. The transcript sequence for AC092718.4.

ENSG00000261061 AC092718.4 (715bp)<br>GCATGGTGGCTCACGCCTATAATCCCAGCACTTTCAGAGGCCAAGGCAGGTGGGTCAAAGGTCAAGAGTTTGA<br>GACTAGCCTTAGCAACATGGTGAAGGTGAACCCCGACTCTACTAAAAATACAAAAATTAGCTGGGCCTGGTGGT<br>GTGCACCTGTAATCCCAGCTACTTAGGAGGCTGAGGCAGGAGAATCGCTTGAACCCGGGAGACATAGGTTGCA<br>GTGAGCCGAGACTGCACCACTGCACTCCAGCCTAGGTGACAGAGTGAGACTCCATCTTAAAAAATAAATAAATA<br>AAATAAAATAAATGACATCACTTTGGTTCAGAGCTCTAAAATGGAGGGAGGAAGCCATTCTAAAAAGGACTCCC<br>TACATGACCTGCAACTTGAAAAAAAATTAAAAGCTCCAAAAAAAAAAACAATACAGGAGCTTACCTTGAACCTT<br>TGAATTGGGCCAAATTGCGATGACCACTGCATCCTGGAAAATTTTATTTCACCAGCACTACAACTCCTCAACAGC<br>ACCAACCAATAAACTATGGATTTTTGTACTAAGCCAGTTGCCTCTTTCAAAACAACTTGTCAACTTGTCTAATCA<br>CCCTCAGCTTTTTTTAAAAACCCCTCCTCTACCCTCTCTCTTCAGAACACAAGTGGCTTCTAGCTGAATCTGTCT<br>CCCAAATTGCAATTCCTAAGACCTCAATAAAAACACCTTGTCTTGCTGC

Supplementary Table 2. The top 107 genes that positively correlated with AC092718.4 in LUAD.

	Statistic	P-value	FDR (BH)	Event_SD	Event_TD
AC092718.4	1	1.00E-72	1.00E-68	515	515
IL4I1	0.682938	5.51E-72	5.51E-68	515	515
SLC2A5	0.680273	3.15E-71	2.10E-67	515	515
HAPLN3	0.65549	1.50E-64	7.49E-61	515	515
SLAMF8	0.634391	2.41E-59	9.65E-56	515	515
GPR84	0.632292	7.56E-59	2.52E-55	515	514
CTSK	0.631386	1.24E-58	3.53E-55	515	515
MSC	0.622449	1.43E-56	3.56E-53	515	515
PLEKHO1	0.620671	3.60E-56	8.00E-53	515	515
IL21R	0.617674	1.70E-55	3.39E-52	515	515
LILRB4	0.617001	2.40E-55	4.35E-52	515	515
ADAM19	0.602785	2.92E-52	4.87E-49	515	515
ALPK2	0.598238	2.63E-51	4.05E-48	515	511
OLFML2B	0.590144	1.21E-49	1.73E-46	515	515
ADAM12	0.589252	1.83E-49	2.44E-46	515	515
NCF1	0.583442	2.65E-48	3.31E-45	515	515
CYTH4	0.579647	1.48E-47	1.74E-44	515	515
COL6A2	0.57525	1.05E-46	1.17E-43	515	515
KIAA1949	0.574579	1.41E-46	1.49E-43	515	515
FERMT3	0.57402	1.81E-46	1.73E-43	515	515
LILRB2	0.574016	1.81E-46	1.73E-43	515	515
VCAM1	0.572478	3.56E-46	3.24E-43	515	515
SLC2A6	0.570835	7.31E-46	6.36E-43	515	515
ARID5A	0.570667	7.87E-46	6.55E-43	515	515
WIPF1	0.570233	9.51E-46	7.52E-43	515	515
TNFRSF9	0.570166	9.78E-46	7.52E-43	515	513
MMP12	0.569378	1.38E-45	1.02E-42	515	515
PTPN7	0.569167	1.51E-45	1.08E-42	515	515
SPHK1	0.567503	3.10E-45	2.14E-42	515	515
ADAMDEC1	0.567134	3.64E-45	2.42E-42	515	514
JAK3	0.563413	1.79E-44	1.15E-41	515	515
FOXP3	0.561873	3.43E-44	2.14E-41	515	515
CD14	0.560314	6.63E-44	4.02E-41	515	515
TNFRSF4	0.559875	7.97E-44	4.69E-41	515	515
LAPTM5	0.559407	9.70E-44	5.54E-41	515	515
EMILIN1	0.557408	2.24E-43	1.24E-40	515	515
THY1	0.556211	3.68E-43	1.99E-40	515	515
LILRB1	0.553845	9.79E-43	5.15E-40	515	515
DOK3	0.55144	2.62E-42	1.34E-39	515	515
WAS	0.551342	2.73E-42	1.36E-39	515	515
TMEM158	0.549269	6.34E-42	3.09E-39	515	515
CPXM1	0.547959	1.08E-41	5.13E-39	515	515
ITGAX	0.547707	1.19E-41	5.54E-39	515	515
LSP1	0.546824	1.70E-41	7.73E-39	515	515
MAFB	0.545333	3.09E-41	1.37E-38	515	515
MMP14	0.545167	3.31E-41	1.44E-38	515	515
LRRC15	0.543262	7.07E-41	3.00E-38	515	515
COL6A1	0.542579	9.26E-41	3.86E-38	515	515
LRRC25	0.541096	1.67E-40	6.79E-38	515	515
RELT	0.539614	2.98E-40	1.19E-37	515	515
PSTPIP1	0.538337	4.92E-40	1.93E-37	515	515
MCHR1	0.537322	7.30E-40	2.81E-37	515	509
SIGLEC10	0.53491	1.86E-39	7.02E-37	515	515
EMP3	0.534235	2.42E-39	8.94E-37	515	515
TWIST1	0.533344	3.40E-39	1.24E-36	515	511
COL6A3	0.533166	3.64E-39	1.30E-36	515	515

PLEKHO2	0.532694	4.37E-39	1.53E-36	515	515
FCGR2B	0.530994	8.36E-39	2.88E-36	515	515
PRRX1	0.530786	9.05E-39	3.07E-36	515	515
ITGB7	0.530413	1.04E-38	3.48E-36	515	515
TNFAIP6	0.528236	2.38E-38	7.80E-36	515	515
CD53	0.527246	3.46E-38	1.12E-35	515	515
NCF1B	0.525056	7.87E-38	2.50E-35	515	514
MXRA5	0.524463	9.82E-38	3.06E-35	515	515
NCF1C	0.524429	9.94E-38	3.06E-35	515	515
LOXL3	0.521806	2.63E-37	7.98E-35	515	515
COL1A1	0.521619	2.82E-37	8.23E-35	515	515
TMEM90B	0.521609	2.83E-37	8.23E-35	515	515
SULF2	0.521602	2.84E-37	8.23E-35	515	515
FCGR1C	0.520892	3.69E-37	1.05E-34	515	515
FCER1G	0.520536	4.21E-37	1.18E-34	515	515
GFPT2	0.519781	5.55E-37	1.54E-34	515	515
CLEC4E	0.518879	7.73E-37	2.12E-34	515	513
PDCD1LG2	0.518729	8.17E-37	2.20E-34	515	515
IL10RA	0.518706	8.24E-37	2.20E-34	515	515
GBP5	0.518026	1.06E-36	2.78E-34	515	515
CD86	0.517779	1.16E-36	3.00E-34	515	515
HS3ST3A1	0.517699	1.19E-36	3.05E-34	515	513
FCGR2C	0.517422	1.32E-36	3.33E-34	515	515
SPI1	0.516703	1.71E-36	4.27E-34	515	515
CILP	0.516548	1.81E-36	4.46E-34	515	515
FCGR1B	0.516153	2.09E-36	5.09E-34	515	515
TNFRSF8	0.515472	2.67E-36	6.44E-34	515	515
GREM1	0.51523	2.92E-36	6.94E-34	515	514
ISLR	0.514163	4.29E-36	1.01E-33	515	515
LCP2	0.513658	5.14E-36	1.20E-33	515	515
SIRPA	0.513116	6.25E-36	1.44E-33	515	515
NETO1	0.512802	7.00E-36	1.58E-33	515	466
COL3A1	0.512794	7.02E-36	1.58E-33	515	515
MEIS3	0.512753	7.12E-36	1.58E-33	515	515
IL12RB1	0.511935	9.55E-36	2.10E-33	515	515
RELB	0.511234	1.23E-35	2.66E-33	515	515
C13orf18	0.511067	1.30E-35	2.80E-33	515	515
FCGR1A	0.510504	1.59E-35	3.38E-33	515	515
CALHM2	0.509756	2.08E-35	4.37E-33	515	515
PCOLCE	0.507993	3.88E-35	8.07E-33	515	515
HCST	0.506634	6.26E-35	1.29E-32	515	515
CORO1A	0.506029	7.74E-35	1.58E-32	515	515
HAVCR2	0.504383	1.38E-34	2.78E-32	515	515
MYO1F	0.503928	1.61E-34	3.23E-32	515	515
WISP1	0.503835	1.67E-34	3.30E-32	515	515
NFAM1	0.503793	1.69E-34	3.31E-32	515	515
KCNK13	0.503684	1.76E-34	3.41E-32	515	514
THBS2	0.502531	2.62E-34	5.04E-32	515	515
ANGPTL2	0.502462	2.69E-34	5.11E-32	515	515
CCL4	0.502101	3.04E-34	5.74E-32	515	515
MATK	0.500151	5.97E-34	1.11E-31	515	515
